# Comparative efficacy and safety of anti-cryptosporidial agents: an *in vitro* study on nitazoxanide, halofuginone lactate, KDU731, and paromomycin against *Cryptosporidium parvum*

**DOI:** 10.3389/fmicb.2024.1463457

**Published:** 2024-10-04

**Authors:** Saffron T. G. Whitta, Bridget Lamont, Rossarin Suwanarusk, Bruce M. Russell, Morad-Rémy Muhsin-Sharafaldine

**Affiliations:** ^1^Department of Microbiology and Immunology, University of Otago, Dunedin, New Zealand; ^2^Department of Parasitology and Entomology, Faculty of Public Health, Mahidol University, Bangkok, Thailand

**Keywords:** *Cryptosporidium*, anti-parasitics, cryptosporidiosis, apicomplexa pathogen, nitazoxanide, halofuginone lactate, lipid kinase inhibitors, paromomycin

## Abstract

This study evaluated the *in vitro* effectiveness of anti-cryptosporidial agents nitazoxanide, halofuginone, the pyrazolopyridine analog KDU731, and paromomycin (PMC) in combating the significant zoonotic pathogen *Cryptosporidium parvum*. The study utilized HCT-8 host cells to culture *C. parvum* and fluorescent microscopy/quantitative PCR (qPCR) for detecting parasitic growth. The efficacy of the compounds was assessed by calculating their inhibitory concentrations (IC) against the total growth of *C. parvum* at 48 h post-infection. The study further investigated the impact of these compounds on early parasitophorous vacuole (PV) formation, merozoite egress, host cell viability, and cell growth cycle. KDU731 displayed the most promising profile, with low nanomolar (102 nM ± 2.28) activity and negligible host cell toxicity. This study offers new insights into the relative efficacy and safety of various anti-cryptosporidial compounds, highlighting their stage-specific effects on *C. parvum* and the consequential impacts on host cells. Identifying safe and effective anti-cryptosporidial agents contributes significantly to the One Health approach, which emphasizes the importance of integrated strategies in controlling zoonotic diseases.

## Introduction

Cryptosporidiosis is a parasitic disease of the small intestine and respiratory tract that is caused by the protozoan parasite *Cryptosporidium* spp., affecting human populations and animals, both domesticated and wild (Innes et al., 2020). The disease is usually self-limiting in immunocompetent patients, with some being asymptomatic. Although varied between patients, the typical symptoms of cryptosporidiosis include severe diarrhea accompanied by abdominal pain and occasional systemic symptoms, such as nausea, low-grade fever, vomiting, loss of weight, and appetite. The symptoms can last up to 2 weeks but can have severe consequences for immunocompromised patients (Smith, 2007). Globally, approximately 84% of all deaths caused by cryptosporidiosis are of children under the age of five (Khalil et al., 2018). Children in developing countries show a greater risk of severe symptoms such as vomiting, dehydration, fever, prolonged diarrhea, and even death due to *Cryptosporidium* infection (Desai, 2020; Khalil et al., 2018). Moreover, as severe symptoms are prolonged in impoverished children, they may cause long-lasting health issues, such as stunted growth and cognitive issues (Beǐer et al., 2006; Guerrant et al., 1999). In addition, *Cryptosporidium*-infected children under the age of one are unable to catch up with lost growth, especially due to the severe malnutrition/infection cycle exacerbating the developmental delay (Caravedo and White, 2023; Theodos et al., 1998). Of all the species that cause cryptosporidiosis in humans, *C. parvum* is of interest due to its high incidence rate and zoonotic transmission between livestock (beef and lamb) and humans (Theodos et al., 1998).

Unfortunately, there is neither a vaccine nor any effective treatment to treat cryptosporidiosis. The antimicrobial thiazolide, nitazoxanide, is the only approved treatment against cryptosporidiosis in humans (Caravedo and White, 2023; Theodos et al., 1998). Although nitazoxanide was initially discovered as a drug for the treatment of tapeworm infection, it was later repurposed as a drug for treating both *Cryptosporidium* spp. and *Giardia intestinalis* infections (Bharti et al., 2021; White, 2004). Studies have suggested that nitazoxanide works by disrupting the parasite’s anaerobic respiration by blocking the pyruvate/ferredoxin oxidoreductase enzyme-dependent electron transfer reaction (Bharti et al., 2021). Although nitazoxanide is efficacious in immunocompetent patients, its efficacy is highly reduced in immunocompromised patients and malnourished children and can be absent in children with HIV (Amadi et al., 2002, 2009; Caravedo and White, 2023).

Animal cryptosporidiosis also poses a significant threat, primarily to livestock farming, with estimated millions in losses due to infection (Gunn and Stott, 1997; Jacobson et al., 2016; Gunn and Stott, 1997; Jacobson et al., 2016; Sweeny et al., 2011). Currently, the only drug for the treatment of cryptosporidiosis in livestock is the synthetic quinazolinone, halofuginone lactate (HFL) (Jarvie et al., 2005; Trotz-Williams et al., 2011). HFL was initially shown to inhibit cancerous tumors and was pushed as a cancer drug, only to be later repurposed as an antiprotozoal drug for the treatment of cryptosporidiosis in livestock (Gill and Sharma, 2022). It has been found that as a prophylactic, HFL can both delay oocyst shedding and reduce symptoms such as diarrhea (Trotz-Williams et al., 2011). However, HFL also causes unspecific toxicity in hosts, with even as little as a double dose being enough to display toxicity symptoms (European Medicine Agency, 2004; Naciri et al., 1993; Silverlås et al., 2009). Furthermore, HFL has also been shown to spread into tissues, such as muscles, organs, and fat, delaying the slaughter of animals, which can further exacerbate the financial burden on farmers (European Medicine Agency, 2004). As HFL does not provide a complete cure for cryptosporidiosis in calves, alternatives need to be examined and further developed (Silverlås et al., 2009).

It is thus of paramount importance that more efficient treatment options to manage *Cryptosporidium* infections are explored. Majuanatha et al. have recently shown that a cryptosporidial lipid kinase inhibitor, KDU731, is able to efficiently kill *C. parvum* in both *in vitro* and *in vivo* systems with minimal toxicity (Manjunatha et al., 2017). KDU731 is a pyrazolopyridine analog that works by competing with ATP molecules by strongly binding with the lipid kinase phosphatidylinositol 4-kinase (PI4K) binding site of *C. parvum* parasites (Manjunatha et al., 2017). More interestingly, KDU731 has been shown to eradicate cryptosporidiosis in an immunocompromised mouse model, giving new hope for cryptosporidiosis treatment among the immunosuppressed population (Manjunatha et al., 2017).

In this study, we directly compared the efficacy of nitazoxanide, HFL, KDU731, and paromomycin (PMC) (as a control) to the asexual growth stages of *C. parvum* using an HCT-8 cells model as the host cells. In addition, the toxicity and host cell life cycle induced by the selected compounds were explored. Our aim was to advance our understanding of the efficacy of the current clinical anti-cryptosporidial compounds (and KDU731) on specific asexual stages of the parasite.

## Methods

### Parasites and host cells

*C. parvum* oocysts (IOWA strain) were obtained from the *Cryptosporidium* production laboratory (University of Arizona, USA) and stored at 4°C in penicillin and streptomycin (100 U/mL and 100 μg/mL, respectively; ThermoFisher Scientific #15140122) in phosphate-buffered saline, pH 7.2 (PBS; ThermoFisher Scientific #21600010), for up to 4 months. The human ileocecal adenocarcinoma host cell line, HCT-8 (ATCC # CCL-225), was a generous gift from Professor Parry Guilford (University of Otago, New Zealand). The HCT-8 cells were cultured and maintained using RPMI 1640 with GlutaMAX™ and HEPES supplements (ThermoFisher Scientific # 72400047) plus 5% fetal calf serum (FCS; Thermofisher Scientific # 10091148). The HCT-8 cells were incubated at 37°C + 5% CO2. When the cells reached 80–90% confluency, they were passaged using 0.25% trypsin–EDTA (Gibco, Cat. #25200056).

### Compounds

Nitazoxanide (Sapphire Bioscience #S1627), HFL (Wuhan Golden Wing Industry & Trade Co., Ltd), KDU731 (Novartis, Singapore), and paromomycin (Sapphire Bioscience #23634) were all reconstituted in 100% dimethyl sulfoxide (DMSO; ThermoFisher Scientific #4121). The reconstituted compounds were stored at −20°C for up to 3 months before use.

### 48-h growth inhibition assay for *Cryptosporidium parvum in vitro* IC value determination

The HCT-8 cells were initially seeded in either 96-well plates or 24-well plates, with 2 × 10^4^ or 1 × 10^5^ of cells/well, respectively. The HCT-8 cells were then incubated until they reached approximately 80% confluency before infection. The *C. parvum* oocysts were first treated with diluted household bleach (at 1:4 in water) for 10 min on ice to sterilize any potential contaminants before being washed with sterile Milli-Q® water and centrifuged twice at 16,000×*g* for 3 min. The final pellet was then resuspended in 100 μL of 0.75% sodium taurocholate (Sapphire Bioscience #16215) and was incubated for 45 min at 37°C + 5% CO2 to allow excystation of the sporozoites. The medium of the host cells was replaced with a fresh medium containing 3% horse serum (R3), and each well plate was supplemented with either 1 × 10^4^ or 1 × 10^5^ sporozoite/well for the 96-well or 24-well plates. The plates were finally spun at 150×*g* for 3 min with low deceleration. The infected cells were then incubated at 37°C + 5% CO2. After 3 h, all wells were gently washed twice with warm PBS, and the medium was resupplied to each well before the incubation was resumed. When resumed, the resupplied medium was supplemented with either nitazoxanide, HFL, KDU731, or paromomycin at specified concentrations.

At specified time points, the infected HCT-8 cells in the 96-well plates were fixed with sterile 4% paraformaldehyde (PFA; VWR Chemicals #28794.295) in PBS for 10 min at room temperature, permeabilized with 0.25% Triton X-100 (Merk #SLBP6453V) in PBS for 10 min at 37°C, and then blocked with 1% bovine serum albumin (BSA; Roche #3853143) in PBS for 1 h at room temperature. The parasitophorous vacuoles (PV) of the parasites were then stained with 2 μg/mL fluorescein-labeled *Vicia villosa* lectin (VVL; Vector Laboratories #ZG0124) in 1% BSA/PBS for 1 h at room temperature, and then, they were nuclei-stained with 2 μg/mL DAPI (Merk #096 M-4014 V) in Milli-Q® water for 15 min in the dark. Following each stain, the wells were washed thrice with 0.1% Tween-20 (Merk #SLBZ8563) in PBS. Each well was then imaged using the Evos FL Auto 2 cell imaging system microscope (ThermoFisher Scientific), and the 25% field view from the center of each well was captured using a 20× magnification lens.

For the infected HCT-8 cells grown in the 24-well plates, the infected cells were first lifted using 0.25% trypsin–EDTA, then centrifuged at 500×*g* for 4 min, and then pellet-resuspended in 200 μL PBS before DNA was extracted using the QIAmp DNA Mini Blood Kit (Qiagen #163043063) as per the manufacturer’s protocol, and the DNA was eluted in 100 μL PBS. The DNA was then subjected to quantitative PCR (qPCR) using the PrimeTime Gene Expression Master Mix (Integrated DNA Technology; IDT #1055772), with the primers for the *C. parvum hsp70* gene at 750 nM each (forward: 5′-AACTTTAGCTCCAGTTGAGAAAGTACTC-3′; reverse: 5′-CAT GGCTCTTTACCGTTAAAGAATTCC-3′; IDT) and for the *hsp70* probe (5′-AATACGTGT/ZEN/AGAACCACCAACCAATACAACA TC-3′; dye/que 6-FAM/ZEN/3′ IBFQ Probe; IDT) at 200 nM. The triplicate samples (at 10 μL) in the MicroAmp Fast Optical 96-well reaction plates (Applied Biosystems #4346906) were then subjected to qPCR using Applied Biosystems ViiA 7 (ThermoFisher Scientific) for 40 cycles with the following thermocycling parameters: initial polymerase activation at 95°C for 15 min and denaturation at 95°C for 15 s, followed by annealing/extension at 60°C for 1 min. To quantify parasitic growth, the standards of the known *C. parvum* sporozoite number (4 × 10^6^–4 × 10^2^) were incorporated into the qPCR plates and used to extrapolate the number of the *hsp70* gene copies from the detected cycle threshold values.

### Early PV formation/trophozoite inhibition assay

As the sporozoites invaded the host cells within seconds of the initial contact, immediate actin polymerization and subsequent PV formation were observed (Guérin et al., 2021). Thus, the HCT-8 cells grown to ≥90% confluency were infected and treated simultaneously with the *C. parvum* sporozoites (2 × 10^4^) and compounds at IC90, respectively. A negative invasion control was performed by pre-fixing the HCT-8 cells with 4% PFA for 10 min at room temperature. The infected cells were incubated at 37°C with 5% CO2 for 3 h to allow early PV/trophozoite formation. The HCT-8 cells were then washed, stained, and imaged following the same protocol as in the standard 48-h growth assay (see above).

### Merozoite egress determination and inhibition assay

This method was adapted from Jumani et al. (2019); briefly, the HCT-8 cells grown to ≥90% confluency were infected with *C. parvum*, as described above. The cells were then infected with 2 × 10^4^ oocysts, post-excystation. To determine when the highest egress of merozoites occurred, 12 time points were taken for the first 24 h of infection and processed for detection via qPCR (see above). Once the merozoite egress time was determined, drugs, at IC90, were added 3 h post-infection (p.i.) and the parasitic growth was measured at the peak egress time point.

### Cytotoxicity assay

The HCT-8 cells were seeded in a 96-well plate and incubated at 37°C + 5% CO2 until the confluency reached 90%. The drugs were added to the wells, and the plate was incubated at 37°C + 5% CO2 for 47 h. In the remaining 1 h, resazurin (Biotium #30025-2) was added at 10% of the volume to the drugged or media-only wells (as a fluorescent background control). After a total of 48 h of incubation, 60% of the wells’ supernatants were carefully transferred into a black-well plate (Greiner, #655076). A VarioskanTM LUX plate reader (Thermo Fisher Scientific #VL0000D0) was used to measure fluorescence at the excitation/emission of 540 nm/585 nm.

### Cell cycle assay

The HCT-8 cells grown in the 24-well plates, until >95% confluency was reached in the R10 media, were treated with the IC_90_ values of the compounds in the R3 for 24 h at 37°C with 5% CO2. At 24 h, the samples were refreshed with the R3 (no phenol red) containing compounds to mimic clinical dosing; dead cells from the supernatants were kept centrifuged (500×*g* for 4 min) and replaced into the culture during the refreshment of drugs. At 48 h, the cells were washed two times with PBS, where the supernatants from the culture and washes were kept in 15 mL falcon tubes. The cells were uplifted gently using Accutase® (ThermoFisher, Cat. #A1110501) at RT for 10 min to be transferred to the falcon tubes. The cells and their supernatants were then spun at 200×*g* for 4 min at 4°C, followed by a 1× cold PBS wash. After another centrifugation, the supernatants were removed and replaced with 1 mL of a hypotonic fluorochrome solution consisting of 1% *w/v* sodium citrate, 0.1% Triton X-100, 50 μg/mL propidium iodide (ThermoFisher Scientific, #P1304MP), and 5 μg/mL of RNase. The samples were stored in the dark at 4°C and were analyzed within 1 week using the BD LSRFortessa Cell Analyzer with the YG_G10/20 channel (50,000 events captured). Flow cytometry data were exported and analyzed using FlowJo v10 after gating for singlet populations; the biology application—Cell Cycle function—was used to obtain the percentage of the cells at the G1 phase, S phase, and G2/M phase of the cell cycle.

### Statistical analyses

Statistical analysis of the experimental results of this project was executed using GraphPad Prism9. All comparisons were made using one-way ANOVA with Šídák’s multiple comparisons test post corrections.

Image J Software Code to detect PVs

Note: *Set the scale before using known distances in μm to pixel values*

Run Macro using the following code: run(“Subtract Background…,” “rolling = 10”);
 run(“8-bit”);
 setAutoThreshold(“Huang dark”);
 //run(“Threshold…”);
 setThreshold(5, 255);
 setThreshold(5, 255);
 //setThreshold(5, 255);
 setOption(“BlackBackground,” false);
 run(“Convert to Mask”); run(“Watershed”);
 run(“Fill Holes”);
 run(“Analyze Particles…,” “size = 2.5–40 summarize”)


## Results

**Figure 1:**
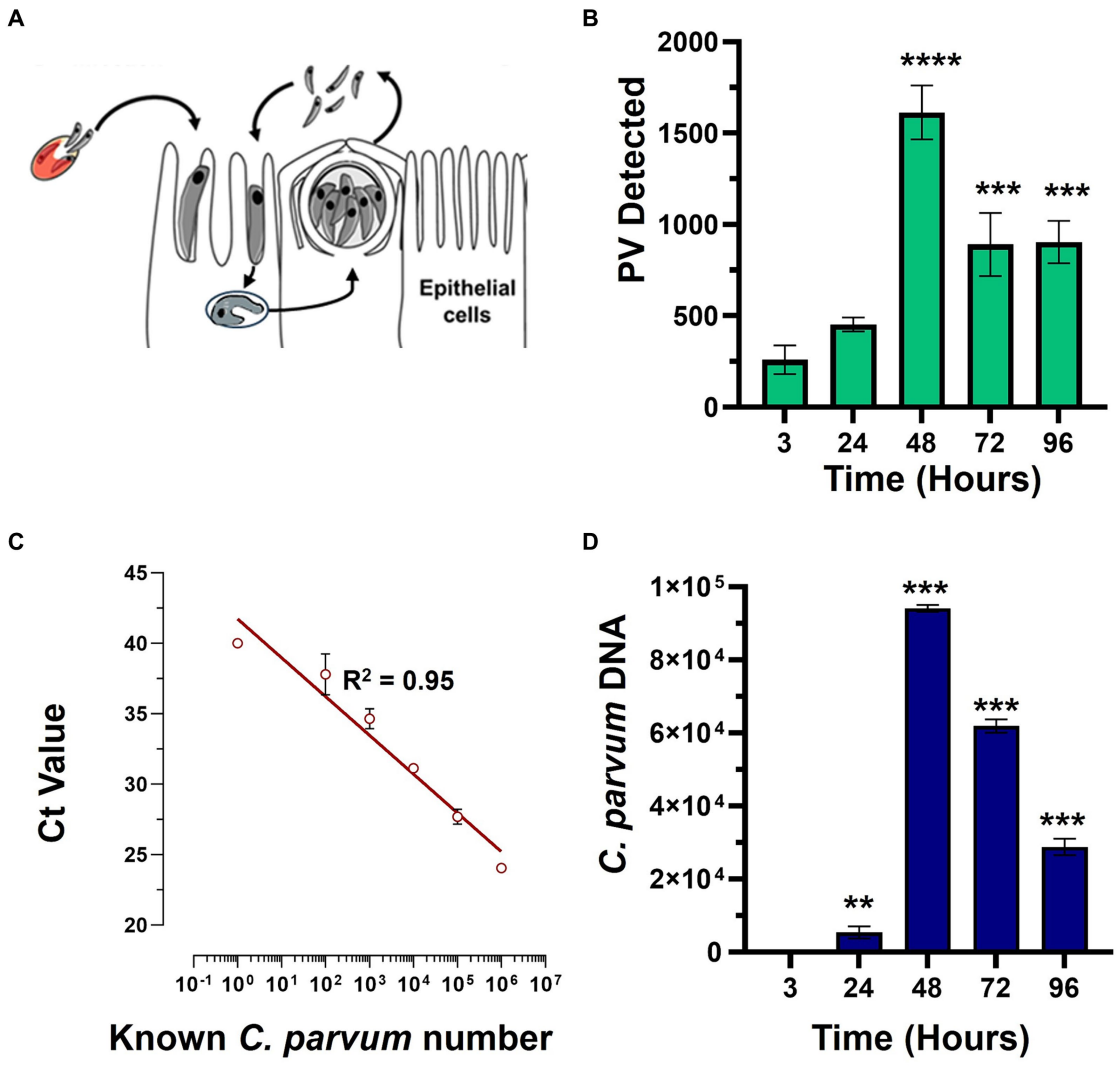
The *in vitro* growth of *C. parvum* in the HCT-8 cells. **(A)** The HCT-8 host cells were infected with the *C. parvum* sporozoites after excystation, and the growth was analyzed at 3-, 24-, 48-, 72-, and 96-h post-infection (p.i.). The samples were either subjected to detection via fluorescent microscopy or qPCR. **(B)** *C. parvum* parasitophorous vacuoles (PVs) were detected using VVL-FITC and quantified using ImageJ. **(C,D)** The *C. parvum-*infected HCT-8 cells were lifted and lysed, and the total genomic DNA was extracted at given time points. The cryptosporidial *hsp70* gene (heat shock protein 70) was targeted using primers and a specific TaqMan probe. **(C)** The representative best-fit graph showing the known parasite numbers against the delta CT (cycle threshold) values; *R*^2^ = 0.9526. **(D)** The unknown numbers of the parasites were interpolated using the best-fit line and CT values. Scale bar: 100 μm. The statistical analysis comparing each time point to the 3-h time point was performed using a one-way ANOVA with Šídák’s multiple comparisons test post corrections. **p < 0.008 and ***p < 0.001.

**Figure 2:**
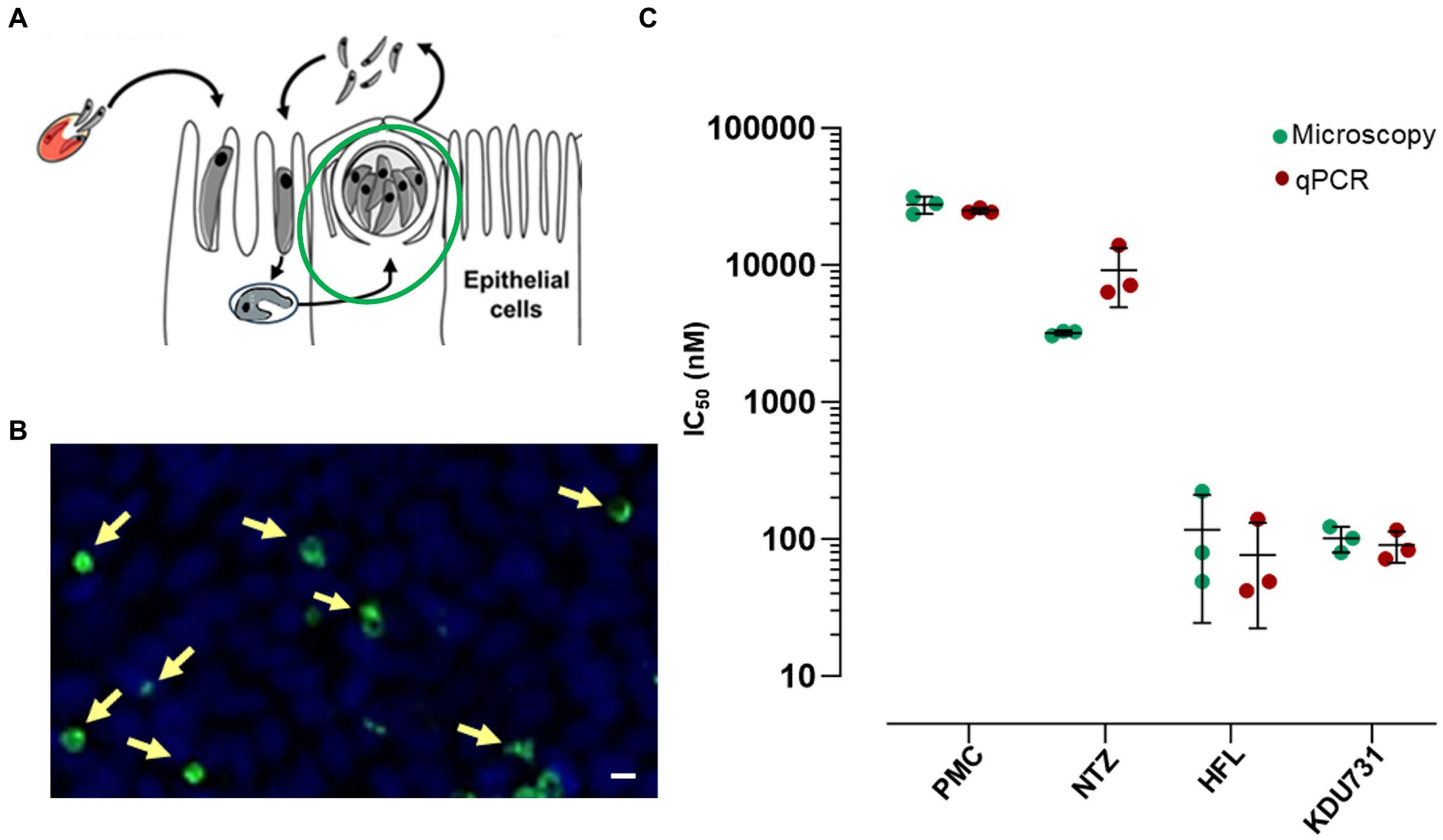
The inhibition of the *C. parvum* growth by the clinical compounds and KDU731 *in vitro* to estimate IC50. **(A,B)** A graphical and representative image of the stage and PV measured 48-h p.i. **(C)** The IC50 values of the parasites treated with each drug were determined either by measuring the PV numbers of the DNA copy number (microscopy or qPCR, respectively) or were calculated by non-linear regression using a four-parameter logistic curve. The IC50 values from each compound were summarized and compared between the two methods. The data are representative of three independent experiments.

This study was conducted to directly compare the *in vitro* efficacy of clinical anti-cryptosporidial compounds nitazoxanide and HFL with the lipid kinase inhibitor KDU731 using paromomycin as a control. The asexual growth of *C. parvum* was first verified by culturing the parasites *in vitro* on the HCT-8 host cells. Either fluorescent microscopy or qPCR was employed to detect the parasitic growth (Figure 1). Compared to the initial parasitic growth (3-h p.i.), the highest parasitic growth was detected at 48 h p.i., with a fold increase of 6.3 (mean PV detected: 1613 ± 146.9 SD; Figure 1B) and 22,089 (mean DNA copy number: 94,099 ± 953.9 SD; Figures 1C,D), for both methods. Then, nitazoxanide, paromomycin, HFL, and KDU731 were all tested against the total growth of *C. parvum in vitro* using the 48-h time point to calculate the half-maximal inhibitory concentrations (IC), IC50 and IC90 (Figures 2A,B). All four compounds demonstrated varying inhibition activities against *C. parvum in vitro*, as verified using both methods of detection. KDU731 displayed the lowest IC values, followed by HFL and nitazoxanide, and finally, the highest IC values were shown by paromomycin (Figure 2C). Overall, the IC50 values did not vary significantly between the samples detected using either microscopy or qPCR, except for nitazoxanide, which displayed a mean difference of 5,563 nM between the two methods (*p* = 0.003; Figure 2C). The IC values for each compound analyzed using microscopy and qPCR are detailed in Table 1. As there were lower variances (SD) between the values detected using qPCR, and the method quantified individual parasites rather than non-specific PVs, the IC values from the qPCR batch were used for the proceeding experiments. The microscopy detection method was, however, chosen for the proceeding experiments as it is significantly less laborious and cost-effective.

**Table 1:** Summary of the anti-cryptosporidial efficacy of the compounds tested.

Drug	Fluorescence microscopy (PV)				qPCR (*hsp70*)			
	IC_50_ (nM)	SD	IC_90_ (nM)	SD	IC_50_ (nM)	SD	IC_90_ (nM)	SD
PMC	**27,720**	2.4	**105,870**	10.92	**25,020**	1.29	**52,250**	4.28
NTZ	**3,607**	3.98	**51,420**	15.81	**9,170**	1	**19,860**	3.22
HFL	**117**	6.34	**195**	15.03	**77**	3.19	**178**	8.39
KDU731	**91**	2.6	**161**	4.99	**102**	2.28	**146**	2.92

PMC, paromomycin; NTZ, nitazoxanide; HFL, halofuginone lactate.

PV, Parasitophorous Vacuole; *hsp70*, Heat-Shock protein-70; *n* = 3.IC, values for selected drugs at 50% or 90% of maximal inhibitory concentration.

**Figure 3:**
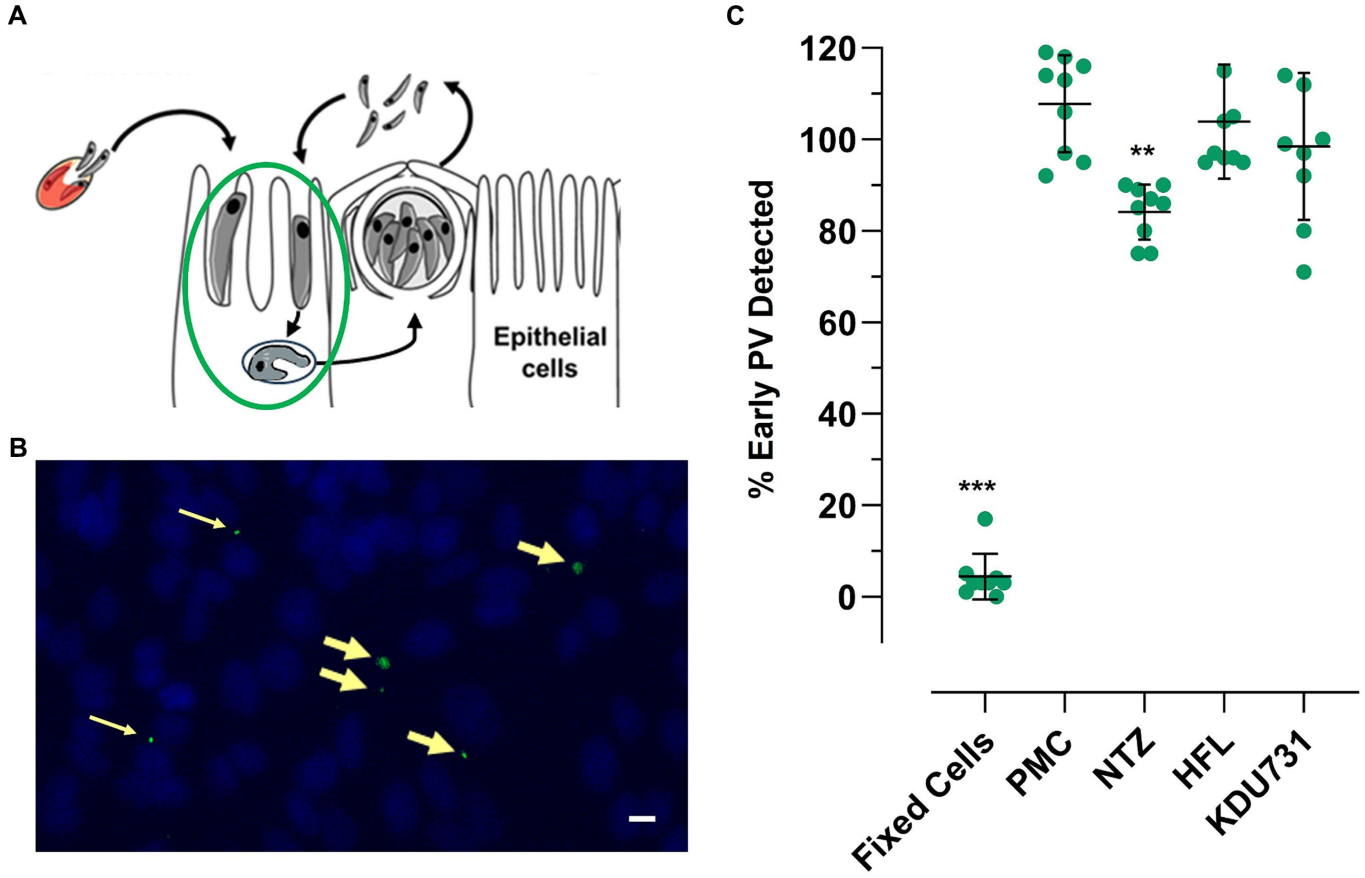
The effects of the selected compounds on early PV (trophozoite) formation. The live or fixed HCT-8 cells were infected with *C. parvum* and treated with each compound at IC90 simultaneously. The cells were fixed with 4% PFA at 3-h p.i. and stained with VVL-FITC (green) and Hoechst 33342 (blue). The cells were analyzed using fluorescent microscopy, and the PVs were counted using ImageJ software. **(A,B)** A graphical and representative image showing early PV formation (yellow arrows). Scale bar = 32 μm **(C)** The quantified PVs were plotted and analyzed using one-way ANOVA with Šídák’s multiple comparisons test post corrections, comparing each to the control (DMSO only) sample. **p = 0.007; ***p < 0.001. The data are representative of three independent experiments.

**Figure 4:**
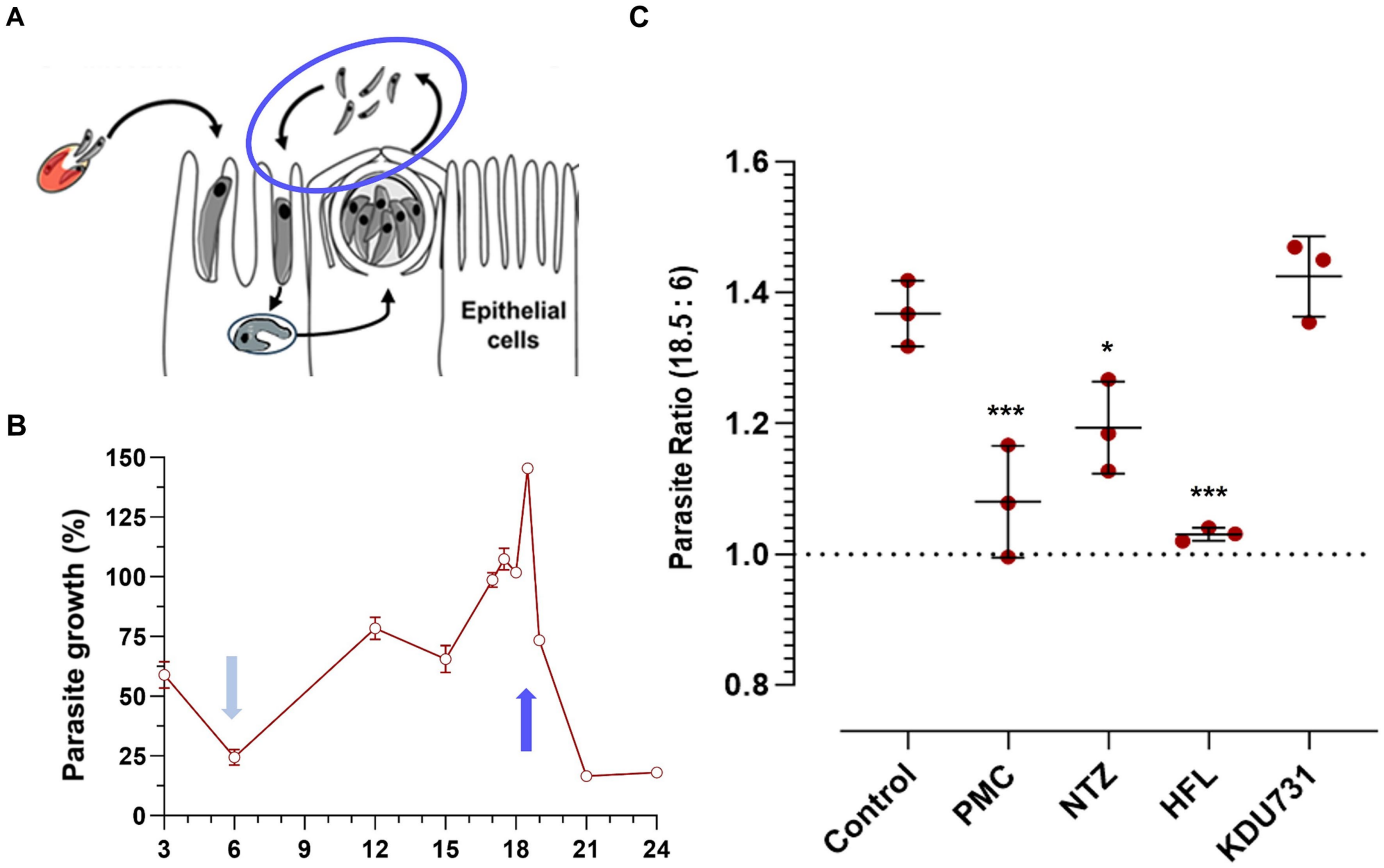
The comparison of the compounds’ effects on the *C. parvum* merozoite egress. **(A,B)** The HCT-8 cells were infected with the *C. parvum* sporozoites and parasites enumerated using qPCR at specified time points. Three peaks were identified at 12-, 17.5-, and 18.5-h p.i. and compared to the lowest yielding time point (6 h). **(C)** The ratio of the parasites detected between the 18.5-and 6-h p.i. samples treated with either of the compounds at IC90. All data were analyzed using one-way ANOVA with Šídák’s multiple comparisons test post corrections, comparing each to the control (DMSO only) sample. *p = 0.02; ***p < 0.001. The results are representative of two independent experiments.

Trophozoites were typically formed 3–4 h p.i. (Figures 3A,B). The PV formed between the sporozoite invasion and trophozoite formation is referred to here as early PV formation. To determine whether any of the test compounds had an effect against the early PV formation, the HCT-8 cells were treated with each compound (at IC90; Table 1). As expected, the control with the PFA-fixed cells was significantly hindered for the early PV formation when compared to the no drug control (4.3% PV detected, ±4.96 SD). This was followed by nitazoxanide, which also significantly suppressed the early PV formation (Figure 3C; mean growth 84.11% ± 6.0 SD). No statistical significance was observed among the other compounds. The next asexual phase of *C. parvum* was the formation and release of merozoites from type I meronts. We cultured *C. parvum* and observed the peaks of parasitic yields within a 24-h period (Figure 4). Using qPCR, the analysis showed that the most observable peaks were at 12-, 17.5-, and 18.5-h p.i., with the 18.5-h p.i. yielding the highest parasitic yield (mean growth 145.4% ± 1.67 SD; Figure 4B). This was next applied using the compounds we were testing, and merozoite burst/egress was studied (Figure 4C). With an untreated mean growth ratio of 1.37, the merozoite egress was significantly suppressed in the parasites treated with halofuginone lactate and PMC, followed by nitazoxanide (mean growth ratio 1.03 ± 0.01 SD, 1.08 ± 0.08 SD, and 1.193 ± 0.07 SD, *respectively*). There was no statistical significance with the parasites treated with KDU731.

**Figure 5:**
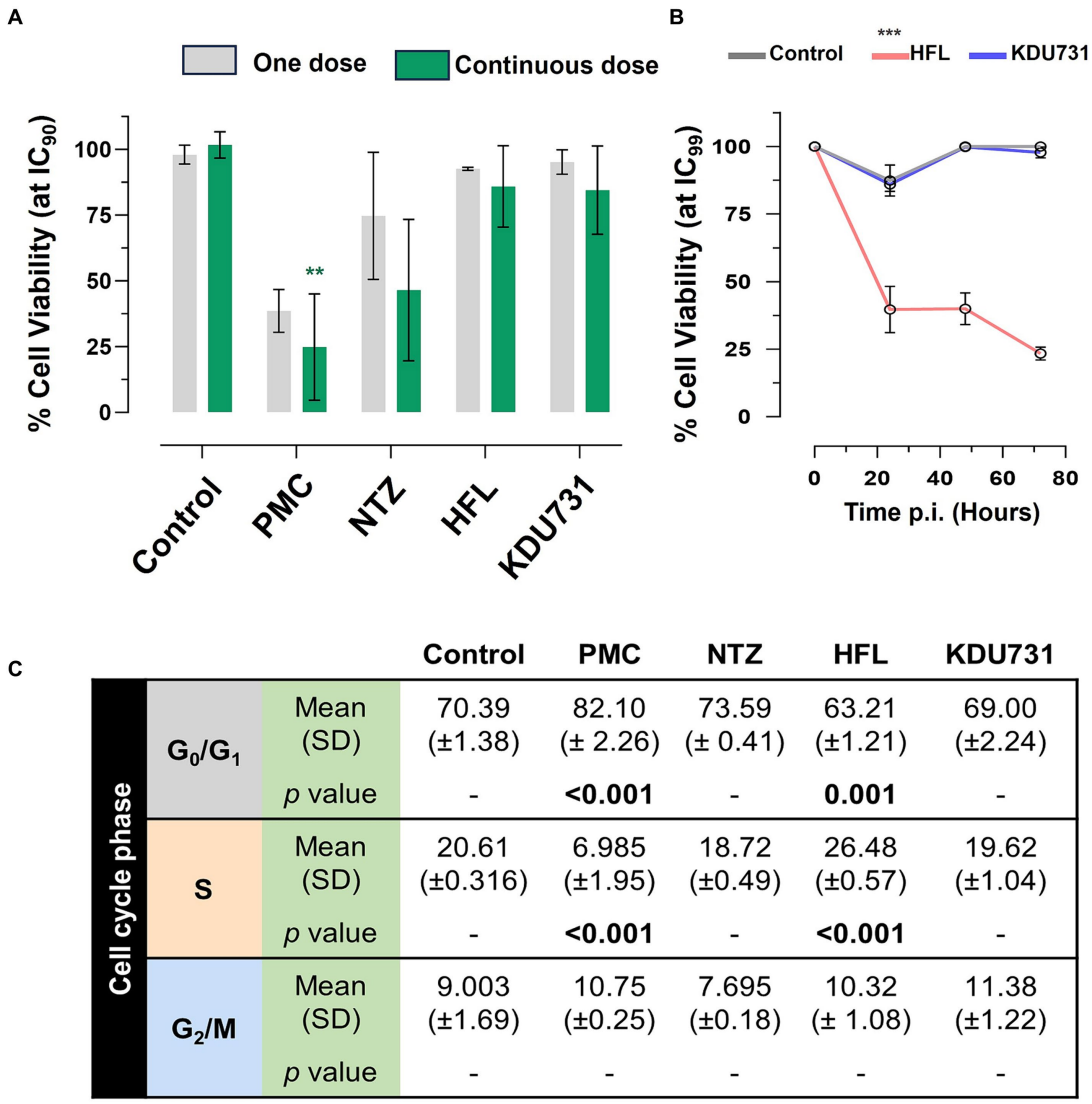
Determining the toxicity of the selected compounds on the HCT-8 host cells using the resazurin assay and cell cycle analysis. **(A)** The HCT-8 cells (at 80–90% confluency) were treated with each compound at IC90 and incubated at 37°C + 5% CO2 for 48 h. The cells were either treated with the compounds once (day zero; one dose) or daily in the fresh medium (continuous dose). At 47-h post-incubation, resazurin was added to the wells and incubated at 37°C for 1 h, and fluorescence was measured (excitation/emission 540/585 nm). **(B)** The cells were instead incubated with either HFL or KDU731 at IC99 (or DMSO only control) for up to 72 h, and the viability was measured using resazurin. The area under the curve for each sample was calculated and plotted. **(C)** Alternatively, after the incubation with all compounds at IC90, the cells were then lifted and strained with a hypotonic fluorochrome solution containing propidium iodide. The cells were then subjected to flow cytometry. Singlet populations were gated, and fluorescence was detected using the YG_G10/20 filter channel (50,000 events captured). The flow cytometry data were analyzed using the FlowJo v10 software, using the Cell Cycle function tool to obtain the percentage of the cells undergoing growth (G1), DNA synthesis (S), and a pre-mitosis growth phase (G2). The mean values from each cycle phase were compared using one-way ANOVA with Šídák’s multiple comparisons test post corrections, comparing each to the control cell population (DMSO only). The results are representative of two independent experiments. PMC, paromomycin; NTZ, nitazoxanide; HFL, halofuginone lactate. All statistical analyses were performed using one-way ANOVA with Šídák’s multiple comparisons test post corrections, comparing each to the control (DMSO only) sample. *p = 0.02; **p = 0.002; ***p < 0.001. The results are representative of three independent experiments.

To determine if the compounds affected the viability of the host cells, the metabolic capacity and growth cell cycle of the host cells were examined. The metabolic capacity was analyzed using a resazurin assay consisting of two treatment regiments: a one-off dose or a continuous daily dose of each compound, all at IC90 (Figure 5A). In both regiments, PMC displayed the lowest cell viability (mean growth one dose: 38.6% ± 8.17 SD; continuous dose: 24.87% ± 20.18 SD). Due to the high variability, nitazoxanide displayed a modest decrease in the host cell viability (mean growth 74.7% ± 24.16 SD) but did not reach statistical significance. There was no significant viability change in the host cells treated with either halofuginone lactate or KDU731. To verify the safety of halofuginone lactate and KDU731, the host cells were treated with both drugs at IC99 for up to 72 h (Figure 5B). There was only a significant drop in the host cell viability when treated with halofuginone lactate compared to the control (*p* < 0.001).

The effects of each drug on the growth cell cycle of the host cells were then investigated (Figure 5C). The drug concentrations selected were from the IC90 concentration because the cells subjected to the IC99 concentration displayed very high toxicity for halofuginone lactate and because there were few cells to analyze. There was a significant increase in the cells undergoing the G1 phase when treated with PMC (mean difference 11.71%) and a significant decrease at the S phase compared to the control (mean difference 13.62% ± 1.95 SD). In contrast, there was a significant decrease in the cells undergoing the G1 phase (mean difference 7.18%) when treated with halofuginone lactate and a significant increase in the cells undergoing the S phase compared to the control (mean difference 5.87%). No statistically significant change was observed among the cells treated with nitazoxanide or KDU731 (Figure 5C).

## Discussion

The findings of this study shed light on the varying efficacies and safety profiles of nitazoxanide, HFL, KDU731, and paromomycin against *C. parvum*. Notably, KDU731 displayed the most promising profile, with low nanomolar activity and low host cell toxicity. This aligned with its mechanism of specifically targeting *Cryptosporidium* lipid kinase PI(4)K (phosphatidylinositol-4-OH kinase), which seemed to have minimal impact on the merozoite egress (Manjunatha et al., 2017). In contrast, nitazoxanide and HFL, while effective in certain stages, showed variability in results and a degree of toxicity to the host cells. It is possible that the positive effects observed by HFL, nitazoxanide, and paromomycin on the merozoite egress were attributed to the subtle toxicity display. As apicomplexan parasites, such as *C. parvum*, are known to hijack the machinery of the host cell for replication (Villares et al., 2020), a damaged host could in turn make the infection inefficient. This emphasizes the importance and critical need for compounds, such as KDU731, which specifically target parasitic factors.

An important distinction was observed in the methodology of parasite detection. Microscopy, measuring PVs, and qPCR, quantifying individual parasites, provided different insights. It is important to note that the qPCR did not account for the viability of the parasite. The decline in the growth of the parasite after the 48-h phenomenon, as detected by both methods, was in agreement with a recent study (Tandel et al., 2019). The HCT-8 *in vitro* model does not allow for the fertilization of cryptosporidial male and female gametes (microgametes and macrogametes, respectively) (Tandel et al., 2019). In particular, nitazoxanide displayed a notable variability between these methods, suggesting potential inconsistencies in its measurement.

The study’s findings on early PV formation and merozoite egress contribute valuable information to the understanding of the compounds’ stage-specific actions. Notably, nitazoxanide significantly suppressed early PV formation, indicating its potential in the early stages of infection. However, its high variability and modest impact on the host cell viability call for caution.

In terms of host cell viability and safety, KDU731 stood out for its minimal toxicity at both IC90 and IC99 concentrations, unlike paromomycin and nitazoxanide. This aspect is crucial as the safety of host cells is a key concern in the treatment of cryptosporidiosis. Furthermore, the study revealed that HFL can alter the host cell cycle, thereby highlighting the superior profile of lipid kinase inhibitors such as KDU731 in terms of safety and minimal impact on host cells.

The clinical drugs nitazoxanide, paromomycin, and halofuginone lactate can also have a negative impact on patients’ intestinal microflora. For example, paromomycin is known to cause significant changes in the gut microflora of patients after treatment (Heinsen et al., 2015). Moreover, a recent study has shown that *C. parvum* infections significantly decrease the alpha diversity of the gut microbiome in humans and mice (Charania et al., 2020; Muhsin-Sharafaldine et al., 2023). Therefore, it is important to consider not only the anti-cryptosporidial effects of the compounds but also the conservation of an optimal gut microbiome profile. The effects of KDU731 on the gut microbiome are yet to be elucidated. However, since it is not a broad-range antimicrobial agent such as paromomycin or nitazoxanide, it is expected to have minimal effects on the microbiome.

Overall, this study emphasizes the need for more efficient and safe treatment options for cryptosporidiosis. The promising profile of KDU731, particularly in terms of efficacy and safety, positions it as a potential leading candidate in the fight against *Cryptosporidium* infections. The findings underscore the importance of considering both the efficacy against the parasite and the safety of the host in developing anti-cryptosporidial therapies.

## Data Availability

The original contributions presented in the study are included in the article/supplementary material, further inquiries can be directed to the corresponding author.
